# Decay of Impact after Self-Management Education for People with Chronic Illnesses: Changes in Anxiety and Depression over One Year

**DOI:** 10.1371/journal.pone.0065316

**Published:** 2013-06-13

**Authors:** M. J. Park, Joseph Green, Hirono Ishikawa, Yoshihiko Yamazaki, Akira Kitagawa, Miho Ono, Fumiko Yasukata, Takahiro Kiuchi

**Affiliations:** 1 Department of Health Communication, Graduate School of Medicine, The University of Tokyo, Tokyo, Japan; 2 Graduate School of Medicine, The University of Tokyo, Tokyo, Japan; 3 Faculty of Social Welfare, Nihon Fukushi University, Nagoya, Aichi Prefecture, Japan; 4 Department of Fundamental Nursing, Fukuoka Prefectural University, Tagawa, Fukuoka Prefecture, Japan; 5 Department of Health Sciences and Nursing, Kawasaki University of Medical Welfare, Kurashiki, Okayama Prefecture, Japan; Federal University of Rio de Janeiro, Brazil

## Abstract

**Background:**

In people with chronic illnesses, self-management education can reduce anxiety and depression. Those benefits, however, decay over time. Efforts have been made to prevent or minimize that “decay of impact”, but they have not been based on information about the decay’s characteristics, and they have failed. Here we show how the decay’s basic characteristics (prevalence, timing, and magnitude) can be quantified. Regarding anxiety and depression, we also report the prevalence, timing, and magnitude of the decay.

**Methods:**

Adults with various chronic conditions participated in a self-management educational program (n = 369). Data were collected with the Hospital Anxiety and Depression Scale four times over one year. Using within-person effect sizes, we defined decay of impact as a decline of ≥0.5 standard deviations after improvement by at least the same amount. We also interpret the results using previously-set criteria for non-cases, possible cases, and probable cases.

**Results:**

*Prevalence*: On anxiety, decay occurred in 19% of the participants (70/369), and on depression it occurred in 24% (90/369). *Timing*: In about one third of those with decay, it began 3 months after the baseline measurement (6 weeks after the educational program ended). *Magnitude*: The median magnitudes of decay on anxiety and on depression were both 4 points, which was about 1 standard deviation. Early in the follow-up year, many participants with decay moved into less severe clinical categories (e.g., becoming non-cases). Later, many of them moved into more severe categories (e.g., becoming probable cases).

**Conclusions:**

Decay of impact can be identified and quantified from within-person effect sizes. This decay occurs in about one fifth or more of this program’s participants. It can start soon after the program ends, and it is large enough to be clinically important. These findings can be used to plan interventions aimed at preventing or minimizing the decay of impact.

## Introduction

People living with chronic health problems can benefit from self-management education [1], [2], [3], [4]. Educational programs aimed at increasing the skills and confidence for self-management can reduce pain, fatigue, disability, anxiety, and depression, and they can improve self-rated general health. Regarding health-related behaviors, the benefits include increases in aerobic exercise and in symptom management [5], [6], [7], [8], [9], [10], [11].

Because the health conditions that these programs address are chronic, attention must be paid to how long the programs’ benefits last. It has been noted that “short-term effects are rarely maintained over long intervals” [12] and that such programs’ “effects tend not to be maintained” [2]. This phenomenon has been given various names: attenuation [8], [13], deterioration [14], relapse [15], backsliding [16], and decay of impact (which is the name we use here) [16]. Decay of impact may not be universal [6], [17], but it has been noted at the whole-group level in many studies [8, 13 14, 15, 18, 19, 20].

For long-term benefits, the decay of impact must therefore be minimized or prevented. To that end, “booster sessions” [1] have been proposed as reinforcements. In the present context, the term “reinforcement” refers to interventions that are intended to help the participants maintain the benefits of the main educational program. These interventions take place after the main program ends. In addition to group-discussion booster sessions [21], [22], reinforcements have also been implemented as an online discussion group to provide peer support [23], an Internet-based program as a follow-up after a face-to-face intervention [24], telephone calls from a health counselor [25], automated telephone calls [26], and printed materials sent to people who had participated in the main program [22], [25]. Studies of reinforcements indicate that they do not consistently provide important benefits (see the Appendix of reference [27]). To explain why they appear to be ineffective, it has been hypothesized that “decay of impact occurs only in a subgroup of these programs’ participants, so any benefits of reinforcements in that subgroup are concealed by whole-group summary statistics.” [27] Here we demonstrate how evidence relevant to that hypothesis can be obtained from a longitudinal cohort study. Specifically, we show how analysis of individual-level data reveals the decay of impact that analysis of whole-group data leaves hidden.

It stands to reason that reinforcements can be optimized on the basis of a clear and accurate understanding of the phenomenon that they are intended to prevent or mitigate. For example, knowledge of when the decay of impact begins could be used to decide when to begin reinforcements [19]. Such basic characteristics of the decay of impact as its timing, prevalence, and magnitude have important practical implications, but those characteristics are unknown because the decay itself has not been an object of study. Therefore, here we also report the prevalence, timing, and magnitude of the decay of impact with regard to two well-defined clinical conditions: anxiety and depression. To the best of our knowledge, no such description of this decay of impact has previously been published.

## Methods

### Participants in the Self-management Program

We analyzed data provided by people with various chronic medical conditions who took part in an educational program aimed at enhancing their ability and confidence to self-manage their chronic illnesses [4], [28]. They were recruited using an announcement on the Internet homepage of the Japan Chronic Disease Self-Management Association [29], and by referrals from flyers left in public service centers. All of the participants were adults, both men and women participated, and they had a wide variety of chronic medical conditions. Socio-demographic information about the participants is shown in Table 1, together with information on their chronic conditions (number of years since diagnosis, numbers of diagnoses, and numbers of participants with each of the most common conditions).

**Table 1:** Demographic and clinical characteristics of the participants in this study (n = 369).

		Number (%)
Age (years)	mean ± SD (range)	49.2±14.0 (19–83)
Sex	Male	73 (19.8%)
	Female	296 (80.2%)
Schooling	High school or less	187 (50.7%)
	College or more	182 (49.3%)
Civil status	Living together with spouse	200 (54.2%)
	Others	169 (45.8%)
Years since diagnosis	mean ± SD (range)	13.6±12.1 (0.5–63)
	median (25%, 75%)	10.0 (4.0, 20.0)
Number of diagnoses	median (25%, 75%)	1.0 (1.0, 2.0)
	min-max	1–7
	1	197 (53.4%)
	2	99 (26.8%)
	3	48 (13.0%)
	≥4	25 (6.8%)
Diagnoses(a)	Allergic disease	92 (24.9%)
	Cardiovascular disease	72 (19.5%)
	Connective tissue disease	67 (18.2%)
	Diabetes	65 (17.6%)
	Rheumatoid arthritis	46 (12.5%)
	Fibromyalgia syndrome	31 (8.4%)
	Cancer	26 (7.0%)
	Depression	22 (6.0%)
	Asthma	16 (4.3%)
	Inflammatory bowel disease	14 (3.8%)
	Parkinson’s disease	12 (3.3%)
	Others	145 (39.3%)

(a) Includes multiple conditions.

### The Self-management Program

The program comprised group-discussion sessions with 5 to 13 participants. The discussions focused on six topics: “1) techniques to deal with problems such as frustration, fatigue, pain and isolation, 2) appropriate exercise for maintaining and improving strength, flexibility, and endurance, 3) appropriate use of medications, 4) communicating effectively with family, friends, and health professionals, 5) nutrition, and, 6) how to evaluate new treatments.” [4] Those topics are introduced in a textbook [30], [31]. Through their discussions, the participants realize how others have experienced and responded to problems similar to their own, even if their diagnoses are different. They talk about how to manage those problems. They learn some self-management skills from the textbook and they also learn from each other. They focus less on what is difficult, and more on what is possible. Then they write specific “action plans” to practice the new self-management skills they learned, and thus they make those skills into new habits.

Each discussion group had 2 lay leaders. Most of the leaders either had a chronic disease or had personal experience with a chronic disease in one of their family members. Their function was not to teach, but to facilitate and manage the discussions, and for that purpose they first underwent structured training. During their training, the trainees “…experience every activity in the workshop’s six sessions, set and report success on their own action plans, practice-teach two activities with a co-leader, and practice handling difficult people in groups” (page 9 of reference [32]). As this self-management program is implemented in Japan, the leader trainees undergo approximately 35 hours of training. For example, a recent training course was held from 9∶00 AM to 4∶30 PM over 5 days. [33]People with different diagnoses attended the same sessions together. There was 1 session each week for 6 consecutive weeks. Between August, 2006 and July, 2011, 87 programs of 6 sessions each were held throughout Japan.

### Measurements

Socio-demographic and other information were obtained via self-administered questionnaires. Those questionnaires also contained the Hospital Anxiety and Depression scale (HADS) [34], [35]. That scale comprises questions about the frequencies of symptoms of anxiety and of depression in the past week. Separate scores were computed for anxiety (7 questions) and depression (7 questions). Possible scores on each question are 0, 1, 2, and 3, and thus the possible total scores on each scale range from 0 to 21. Higher scores indicate more symptoms and more frequent symptoms, i.e., more distress. In the present study, Cronbach’s coefficient alpha was 0.84 for the anxiety scale and 0.75 for the depression scale. Those values are typical for these scales [26], and they are nearly the same as those reported from a previous study in Japan [34].

### Study Design and Timing of Measures

**Figure 1:**
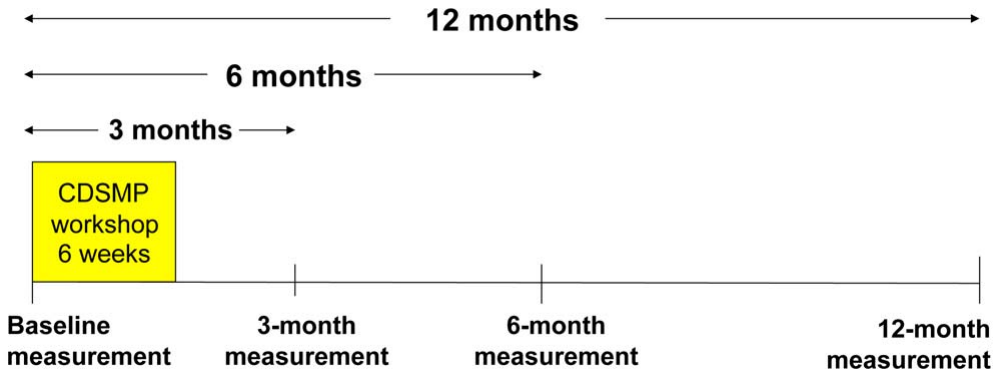
Timing of data collection. Data were collected once before the educational program began (baseline), and then three more times over the following year.

This was a longitudinal cohort study in which data were collected four times over one year. Baseline data were collected before the first group-discussion session, and follow-up questionnaires were sent by postal mail 3 months, 6 months, and 12 months later (Figure 1). A self-addressed post-paid envelope was included. If a follow-up questionnaire was not returned within two weeks, a reminder postcard was sent.

### Definition and Interpretation of Decay of Impact

We used two different definitions of decay of impact, a distribution-based definition and an anchor-based definition. These two are described separately below.

#### 1. Distribution-based definition

To operationalize the concept of decay of impact, we used an index of change similar to Cohen’s d [37], which is a standardized effect size. This method of quantifying change was previously used in a very similar context. Specifically, Nolte, et al. [38] studied adults who had chronic medical conditions and participated in self-management educational programs. They measured emotional well-being from the responses to questions regarding “overall health-related negative affect; attitude to life; anxiety, stress, anger and depression.” That study is similar to the present study in terms of the population, the intervention, one of the outcome measures, and the focus on changes in individuals. They quantified each individual’s change by using a within-person individual effect size: “The within-person individual ES {effect size} was defined as the individual change score divided by the standard deviation of the baseline score of the sample” [38], [39]. Each of those changes was considered to be “substantial” if it was one half standard deviation or greater. The criterion of one half standard deviation was chosen because previous work regarding effect sizes shows that it “approximates a minimal important difference” [38]. As a criterion for the minimal important difference in many patient groups and for many outcomes, the half standard deviation criterion has a “remarkable universality” [40], [41].

For each participant, Nolte, et al. analyzed one baseline value and one follow-up value, whereas in the present study we analyzed one baseline value and three follow-up values. For each participant, they identified substantial change by applying the half standard deviation criterion once, whereas we applied it twice (as described below). We considered a participant to have decay of impact if that participant’s data met both of the following two conditions: (1) there was substantial improvement after the baseline value was measured, and (2) there was substantial decline after that substantial improvement.

First, using the effect size and the half standard deviation criterion for substantial change, we identified the participants who had substantial improvement, whether it was evident at 3 months or at 6 months (or both). For that step, each participant’s change score was the difference between the baseline score and the best of the two intermediate follow-up scores (either the score at 3 months or the score at 6 months, whichever was lower, i.e. whichever indicated less distress).

Next, including only the participants who had substantial improvement, we applied the same “half a standard deviation” criterion again. Specifically, for each participant who had substantial improvement we computed the difference between the best score and the 12-month score, and then we divided the resulting individual change score by the standard deviation of the scores at the time of the best score. Among the participants who had substantial improvement (whether at 3 months or 6 months), those whose 12-month score was at least half a standard deviation higher (i.e., worse) than their best score were said to have decay of impact, because they had substantial decline after their substantial improvement.

#### 2. Anchor-based definition

The half standard deviation criterion is a distribution-based criterion for defining a minimal important difference. In addition to the results obtained using that method, we also report the results obtained using an “anchor-based” method [42]. Anchor-based methods depend on an “association between the targeted concept of a PRO {patient-reported outcome} instrument and the same or closely related concept measured by an independent anchor or anchors.” [43] They have advantages and disadvantages. The advantage of anchor-based methods is that they are strict. According to Cappelleri and Bushmakin [44], “The chosen anchor should be clearly understood in context and be easier to interpret than the PRO measure of interest, and the anchor should be appreciably correlated with the targeted PRO.” Therefore, for the present study an anchor would be appropriate only if it were easier to interpret than the HADS and appreciably correlated with the HADS. It would also need to have “intuitive meaning” [42], and it would have to be measured independently of the HADS. The disadvantage of anchor-based methods is that, because they must meet such strict requirements, good anchors are rare. No such anchor was available within the present study. A search of the literature revealed one report of an anchor-based criterion for HADS scores: Puhan, et al. [45]. All of the participants in that study were patients with chronic obstructive pulmonary disease who were in a rehabilitation program. In that study the anchor was the Chronic Respiratory Questionnaire [46], and the criterion for a minimal important difference was 1.5 points on the each of the HADS subscales (Table 2 of reference [45] and the Conclusion of reference [45]). However, the population and the intervention were different from those in the present study. Furthermore, in that study the criterion for HADS scores was tied to results on the Chronic Respiratory Questionnaire (Table 2 of reference [45]), but in the present study most of the participants did not have chronic respiratory disease (Table 1).

**Table 2:** Questionnaire return and non-return at each follow-up time.

	Baseline	3-month follow-up	6-month follow-up	12-month follow-up
Returned(a)	643	492	470	458
Not returned(b)	0	151 (23.5%)	173 (26.9%)	185 (28.8%)

(a) Number of questionnaires returned at the time indicated.

(b) Number (%) of questionnaires not returned at the time indicated.

Because no anchor was available within the present study, and also because of the differences between the present study and the study by Puhan, et al. [45], we consider the distribution-based criterion (i.e., half standard deviation) to be the most appropriate criterion for this study. Nonetheless, below we also report results obtained using Puhan, et al.’s anchor-based value. This is done for the sake of completeness, as 1.5 seems to be the only anchor-based criterion reported for the HADS. It is also done so that those results will be available for future analyses of relations between distribution-based and anchor-based methods in general. By the criterion of Phuan et al., a participant was considered to have decay of impact if both of the following two conditions were met: (1) there was an improvement of at least 1.5 points after the baseline value was measured, and (2) there was a decline of at least 1.5 points after that improvement.

### Analyses of Decay of Impact

#### Prevalence

For anxiety and depression separately, we computed the percentage of participants who had decay of impact. Prevalences are expressed as percentages of the group as a whole, and also as percentages of those who initially had substantial improvement.

#### Timing

For each participant who had decay of impact, the time at which the decay began was estimated as the time of that participant’s lowest (i.e., “best”) score. To be able to distinguish those participants in whom the decay began relatively early from those in whom it began later, we included data only from participants who returned all four questionnaires.

#### Magnitude

For each instance in which the definition of decay of impact was met, we defined the magnitude of the decay as the difference between the best value (at 3 months or at 6 months) and the last measured value (at 12 months). Magnitudes of change are shown in HADS-scale units (minimum 0, maximum 21 for each scale), and also in standard-deviation units. Cumulative frequency distributions [43] of results from all participants who had substantial improvement at 3 or 6 months are also shown.

The magnitude of anxiety and of depression can also be analyzed in terms of clinical categories. Here we used the cutoffs that were previously used in Japan to define non-cases (scores less than 9), possible cases (scores of 9, 10, and 11), and probable cases (scores greater than 11) [34]. Separately, we also used “8 or above” as a definition “caseness,” based on the work of Bjelland et al. [36]. Using those cutoff scores to define clinical categories, we counted the number of participants with decay of impact who moved from one clinical category to another as their status first improved and then declined over the course of the study.

We also compared the magnitude of the decay of impact to the magnitude of previously-reported differences between known groups: university students and psychiatric outpatients in Japan [34]. Their mean scores on the anxiety and depression scales were, respectively, 6.5 and 5.9 for the students, and 8.3 and 8.2 for the outpatients. Thus, the university students differed from the psychiatric outpatients by 1.8 points on the anxiety scale and by 2.3 points on the depression scale, and we compared those differences to the magnitude of the decay of impact.

### Software

Statistical analyses were done with Microsoft Excel (version 14.2.3) and IBM SPSS version 20.

### Ethical Considerations

This study was approved by the Research Ethics Committee of the Graduate School of Medicine at the University of Tokyo (IRB document number 1472). Participation in the program and in this research were voluntary. Informed consent was obtained in writing from all participants before the study began.

## Results

### Participants

Among the 643 people who took part in the self-management program, 369 returned all four of the questionnaires. Data from those 369 people were used in this study. The numbers of questionnaires returned and not returned at each follow-up time are given in Table 2. Patterns of questionnaire return are shown in Table 3. There was no significant difference between the participants who returned all of the questionnaires and those who did not return one or more of the questionnaires, with regard to the number of diagnoses, or with regard to the depression score at baseline (Table 4). The mean anxiety score at baseline was 1.05 points higher among those who did not return one or more of the questionnaires (Table 4). The effect size (Cohen’s d) for that 1.05-point difference was 0.24, which would generally be considered to be small [37].

**Table 3:** Eight patterns of questionnaire return.

Baseline	3-month follow-up	6-month follow-up	12-month follow-up	Number of participants	Percent of total
Yes	Yes	Yes	Yes	**369** (a)	57.4%
Yes	Yes	Yes	No	52	8.1%
Yes	Yes	No	Yes	31	4.8%
Yes	No	Yes	Yes	35	5.4%
Yes	Yes	No	No	40	6.2%
Yes	No	Yes	No	14	2.2%
Yes	No	No	Yes	23	3.6%
Yes	No	No	No	79	12.3%

Yes: questionnaire returned; No: questionnaire not returned.

(a) Data from these 369 participants were used in this study. Only data from participants who returned all four questionnaires were used, because those were the only participants regarding whom it was possible to determine, for each individual who had decay of impact, whether that decay began at 3 months or at 6 months after the baseline measurement.

**Table 4:** Comparison of those who returned all 4 questionnaires and those who returned fewer than 4.

	All who were eligible for the study(a)	Those who returned all 4 questionnaires	Those who returned fewer than 4 questionnaires
	(n = 643)	(n = 369)	(n = 274)
Number of diagnoses	1 (1, 12)(b)	1 (1, 7)	1 (1, 12)
Anxiety score at baseline	6.99±4.42	6.54±4.08	7.59±4.78
Depression score at baseline	7.35±3.96	7.23±3.90	7.53±4.01

Tests for differences between those who returned all 4 questionnaires and those who returned fewer than 4:

For the number of diagnoses, *U* = 50464.5, *p* = 0.646, *r* = 0.02.

For anxiety scores at baseline, *t*(530) = 2.93, *p* = 0.003, *d* = 0.24 (*df* adjusted due to unequal variances).

For depression scores at baseline, *t*(640) = 0.94, *p* = 0.344, *d* = 0.07.

(a) The people who were eligible for the study were adults who had at least one chronic medical condition and took part in an educational program to enhance their ability and confidence to self-manage their chronic condition(s).

(b) Median (minimum, maximum). The minimum is the same as the median because many people (336, 52.3%) had only one diagnosis. For the distribution of number of diagnoses among all who were eligible for the study, skewness was 2.49.

Approximately four fifths (80.2%) of the participants were women, and they ranged in age from 19 to 83 years (mean age: 49). Approximately half of them had schooling at or above the college level (49.3%), and slightly more than half were living together with a wife or husband (54.2%). Almost half of them reported having more than one diagnosis (46.6%). Socio-demographic and clinical details are given in Table 1.

### Anxiety and Depression at Baseline

On both anxiety and depression, the scores at baseline covered almost the entire available range (Table 5). On anxiety, 30.4% of the 369 participants had scores of 9 or higher at baseline, which means that they would be classified as either possible cases or probable cases. On depression that percentage was slightly higher: 38.2%.

**Table 5:** Anxiety and depression scores at baseline, and numbers of participants in the three clinical categories (n = 369).

	Anxiety	Depression
Mean ± SD	6.55±4.08	7.23±3.92
Median (25%, 75%)	6.0 (3.0, 9.0)	7.0 (4.0, 10.0)
Minimum-maximum(a)	0–18	0–19
Clinical category(b)		
Probable case (score >11)	49 (13.3%)	56 (15.2%)
Possible case (score = 9, 10, or 11)	63 (17.1%)	85 (23.0%)
Non-case (score <9)	257 (69.6%)	228 (61.8%)

(a) The lowest possible score is 0 and the highest possible score is 21. Lower scores indicate fewer symptoms and less frequent symptoms.

(b) These are the categories used by Matsudaira, et al. [34].

### Short-term Changes in Anxiety and Depression

By the time of the first follow-up measurement, which was 3 months after the baseline measurement, anxiety had substantially worsened in 16.3% (60/369) of the participants and it had substantially improved in 27.4% (101/369). In the remaining 56.4% (208/369) there was no substantial change. For depression those percentages were 24.4%, 36.8%, and 38.8%, respectively (90, 136, and 143 of 369).

### Prevalence of Decay

#### Anxiety

Almost 40% of the participants had substantial improvement in anxiety at 3 months, 6 months, or both (146/369). Decay of impact occurred in 19% of the whole group (70/369). Those 70 participants were 48% of the 146 who had previously had substantial improvement.

#### Depression

Half of the participants had substantial improvement in depression at 3 months, 6 months, or both (189/369). Decay of impact occurred in 24% of the whole group (90/369). Those 90 participants were 48% of the 189 who had previously had substantial improvement.

With Puhan, et al.’s [45] criterion of 1.5 points (rather than the half standard deviation criterion), the prevalence of decay of impact on anxiety was slightly higher (24%, 87/369), and on depression it was essentially the same as with the half standard deviation criterion (25%, 91/369).

Some of the participants had decay of impact on anxiety only and some had it on depression only, but 31 of them had it on both of the measures: i.e., 44% of those with decay on anxiety and 34% of those with decay on depression had decay on both measures.

### Timing of Decay

Regarding timing, the best score was the 3-month value in about one third of the participants who had decay of impact (anxiety: 30%, 21/70; depression 38%, 34/90).

### Magnitude of Decay

**Figure 2:**
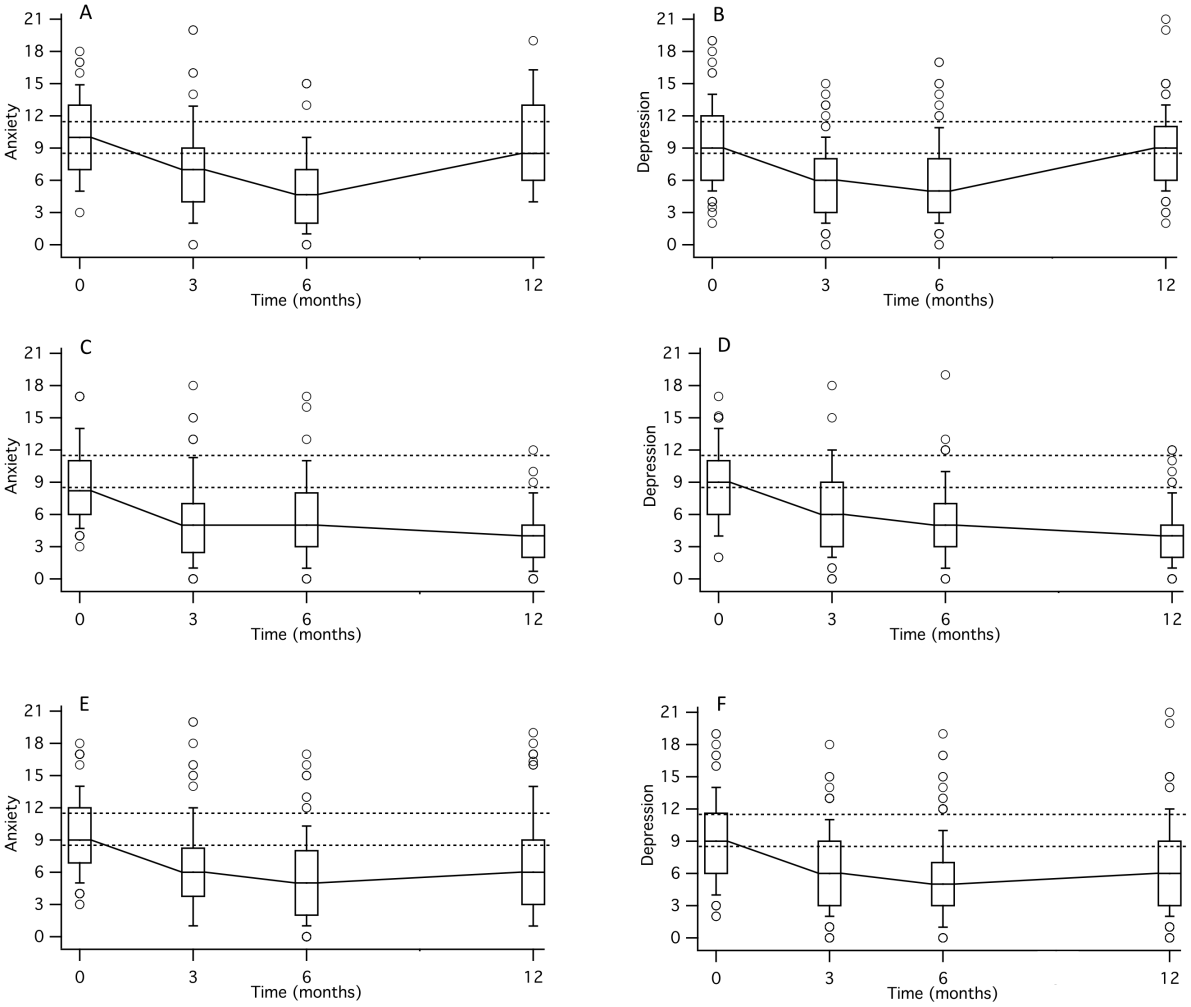
Box-and-whisker plots showing scores on the anxiety and depression scales at the four measurement times. The line inside each box indicates the median, the bottom and top indicate the 25th and 75th percentiles, and the ends of the whiskers indicate the 10th and 90th percentiles. Lines connecting the medians were drawn to show patterns of change over time. Higher scores indicate more symptoms and more frequent symptoms. The horizontal dotted lines show the criteria used to define non-cases (scores ≤8), possible cases (scores of 9, 10, and 11), and probable cases (scores ≥12) [34]. Panels (A) and (B) show the results from the participants who had substantial improvement that was followed by substantial decline. The decay-of-impact pattern is clearly visible: First the scores decreased (improvement) and later they increased (worsening). It is also clear that, as part of the decay of impact, many participants moved among the clinical categories. Specifically, many of them moved into a better clinical category within the first half of the follow-up year and by the time of the last measurement they had returned to a worse category. For (A), n = 70, and for (B), n = 90. Panels (C) and (D) show the results from the participants who had substantial improvement that was *not* followed by substantial decline. These participants did not have decay of impact. For (C), n = 76, and for (D), n = 99. Panel (E) shows the data from (A) and (C) combined (n = 146), and panel (F) shows the data from (B) and (D) combined (n = 189). When the data are combined the decay of impact is almost completely obscured.

The decay-of-impact pattern is easy to see in the results of the within-person analyses (Figures 2A and 2B) but not in the whole-group summaries (Figures 2E and 2F). By definition, the smallest possible decay of impact was half a standard deviation, so the frequency distributions of those magnitudes were truncated at 0.5 and were right-skewed (Table 6). The median magnitudes of the decay on anxiety and on depression were similar: 4 points, which was about 1 standard deviation (Table 6).

**Table 6:** Magnitude of decay of impact.

		Anxiety (n = 70)	Depression (n = 90)
HADS score units			
	Mean ± SD	5.64±3.43	4.65±2.58
	Median	4.0	4.0
	(25%, 75%)	(3.0, 8.0)	(3.0, 6.0)
	Skewness	0.94	1.13
Standard-deviation units			
	Mean ± SD	1.47±0.90	1.19±0.66
	Median	1.1	1.0
	(25%, 75%)	(0.8, 1.9)	(0.7, 1.6)
	Skewness	1.01	1.12

**Figure 3:**
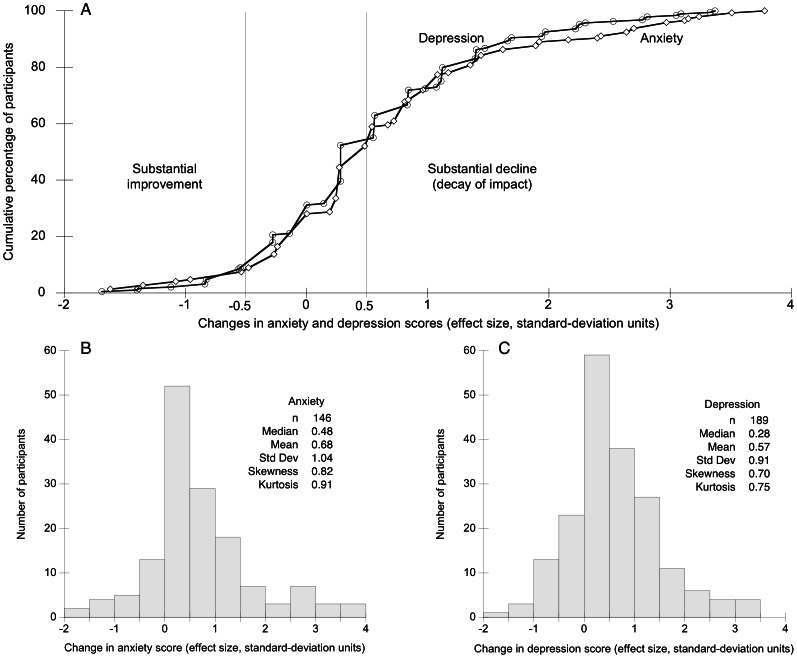
Cumulative frequency distributions and histograms of changes from the time of the best score to the end of the follow-up year. The cumulative frequency distributions in panel (A) show changes in scores on the anxiety and depression scales. On these scales, higher scores indicate more distress. Thus, change scores less than zero indicate improvement and change scores greater than zero indicate decline. Changes are shown in standard-deviation units. Vertical lines indicate half a standard deviation above and below zero. By the definition used in this study, increases of half a standard deviation or more were considered to indicate substantial worsening of anxiety or depression. These results are from participants who had substantial improvement between baseline and 3 months, between baseline and 6 months, or both: n = 146 for anxiety, and n = 189 for depression. The changes shown occurred between the time of the best score (i.e., at 3 or at 6 months) and the end of the follow-up year. Thus, points to the right of the vertical line at +0.5 indicate substantial decline in participants who had previously had substantial improvement, i.e. decay of impact. Displays such as (A) illustrate the full range of the measured changes, and allow one to easily see the proportion of participants in whom the magnitude of change meets or exceeds any given value. The histograms in panels (B) and (C) were constructed from the same data used to construct the cumulative frequency distributions in panel (A). Both histograms show distributions that are slightly positively skewed.

The cumulative frequency distributions show results obtained from all of the participants who had substantial improvement at 3 months or 6 months, whether or not they later had decay of impact (Figure 3). These curves show the distribution of changes measured from the time of the best score to the end of the follow-up year, to illustrate the full range of changes measured over that time.

During the follow-up year, many participants with decay of impact moved among the three clinical categories of non-case, possible case, and probable case. For both anxiety for depression, about half of them moved into a less severe category early in the follow-up year, and about half of them moved into a more severe category by the time of the 12-month measurement. Specifically, between the time of the best score and the time of the 12-month score, 50% (35/70) of those with decay of impact moved into a more severe anxiety category and 48% (43/90) of them moved into a more severe depression category. As judged by the criterion of a score of 8 or higher for clinical “caseness” [36], in more than half of the participants who had decay of impact that decay involved a change in status from non-case to case: 67% (47/70) for anxiety, and 62% (56/90) for depression.

Regarding known groups, in Japan, university students differed from psychiatric outpatients by 1.8 points on the anxiety scale and by 2.3 points on the depression scale [34], which were approximately half as large as the decay of impact in the present study: The median magnitude of the decay was 4 points, both on anxiety and on depression (Table 6).

## Discussion

### Decay of Impact: Main Findings

The within-person analyses revealed patterns that were almost completely obscured by whole-group summaries (compare Figure 2A with 2E, and Figure 2B with 2F). This is consistent with the hypothesis that decay of impact occurs only in a subgroup of these programs’ participants [27].

The main findings regarding prevalence, timing, and magnitude of the decay of impact are as follows. First, about 40%–50% of all of the participants had substantial improvement within the first six months, and, both on anxiety and on depression, about half of those who had substantial improvement later had substantial decline. This is also consistent with the hypothesis that decay of impact occurs only in a subgroup. Second, in about one third of the participants the decay began 3 months after the baseline measurements. Third, on both anxiety and depression the median magnitude of the decay of impact was 4 points, which was about 1 standard deviation. Many participants first moved into less severe clinical categories, but later “decayed” back into a more severe clinical category.

### Timing of Decay of Impact

Because no previous studies have focused on the decay of impact after this type of educational program, it is not possible to compare the timing of decay found here with previous findings. However, some studies have been done in related areas. Krebs et al. [18] meta-analyzed a total of 88 studies of computer-tailored interventions intended to influence various health-related behaviors (smoking cessation, dietary fat reduction, increasing fruit and vegetable intake, physical activity, and mammography screening). They found large variation between studies, but overall there was a decay of impact that began 4 to 6 months after the baseline measurements (see Figure 1 in reference [18]), which is generally consistent with the present results. Hennessy et al. [19] studied the timing of the decay of impact after an intervention to increase self-efficacy for condom use to prevent HIV infection. Their goal was to determine when reinforcement should be given, and they found that the decay of impact began less than 3 months after the end of the initial intervention. That finding is also consistent with the implication of the present findings, which is that reinforcements should start early.

### Interpreting the Magnitude of the Decay of Impact

One basic question about the decay of impact is whether it is large enough to merit further attention. When using Cohen’s d [37] or a similar effect size, it is common to interpret effects of half a standard deviation or larger as important [40], [41], [43], and by that criterion every instance of decay reported here was important, but of course that follows (tautologically) from the operational definition of decay of impact that we used.

Another approach to interpretation involves cutoff points that define clinical categories. This approach is not ideal, because there may be disagreement about the most appropriate number of categories and about clinical “gold standards” for anxiety and for depression. Another disadvantage is that categorization always causes loss of information [47]. Nonetheless, we discuss categories of HADS scores here because they are used so commonly.

When clinical categories are used, it would be reasonable to regard an instance of decay of impact as clinically important if it entailed a change in status from a less severe to a more severe category. Various cutoff points defining clinical categories have been proposed [35], [48]. The cutoffs that were previously used in Japan define three categories: non-cases, possible cases, and probable cases [34]. As noted above, by those criteria, the decay was clinically important in about half of the participants, both on anxiety (50%) and on depression (48%).

The results were slightly different when two clinical categories were used rather than three. Bjelland et al. [36] defined two categories. They reviewed 24 studies in which HADS results were compared with diagnoses “made by a structured or semistructured diagnostic interview.” They found that for both anxiety and depression scores “in most studies an optimal balance between sensitivity and specificity was achieved when caseness was defined by a score of 8 or above” [36]. Thus, if the decay of impact entailed an increase from a score below 8 to a score of 8 or higher, then it would be judged to be a clinically important worsening (that is, a change in status from non-case to case). As noted above, by that criterion, the decay was clinically important in more than half of the participants, both on anxiety (67%) and on depression (62%).

Therefore, whether the number of clinical categories used was two [34] or three [36], it is clear that there was clinically important improvement followed by clinically important worsening in half or more of those who had decay of impact. That finding may underestimate the importance of the decay. Bennette and Vickers [47] illustrate how information can be lost when data are categorized by quantiles, and the same point can also apply to categorization by other criteria. For example, if a person’s HADS depression score increases from 12 to 20 that person would not move from a less severe to a more severe category, although the clinical worsening might be very important. It is important to remember that the utility of clinical categories is limited. In the present context, important declines could be overlooked if the decay of impact is viewed only as movement between categories.

Yet another approach to judging the importance of the decay of impact is to compare its magnitude with the magnitude of the difference between known groups. We were able to make such a comparison using results from a study of university students and psychiatric outpatients in Japan [34]. In that study, those two groups differed by 1.8 points on the anxiety scale and by 2.3 points on the depression scale. In the present sample of adults in Japan who had chronic medical conditions, the median magnitude of the decay of impact was 4 points on both scales (Table 6). Therefore, the decay was approximately twice as large as the difference between university students and psychiatric outpatients.

### Operational Definitions of Decay of Impact

To define the decay of impact, in this study we used a distribution-based method incorporating the “half a standard deviation” criterion. No appropriate anchor-based criterion was available from within the study. Also, the half standard deviation criterion is known to be widely applicable [40], [41], [43]. This method was used previously to define “substantial” changes in a similar outcome (emotional well-being) in a similar population that underwent a similar intervention [38].

As an example of a different definition of decay of impact, in one study relapse was defined by using a disease-specific measure that included self-identification as a “relapser” [15]. Another possibility would be to estimate the standard error of measurement, which depends on estimates of score reliability [43], [49], although that criterion is used less commonly than the half standard deviation criterion [50]. Yet another possibility would be to do a separate study to find, for minimal important changes in HADS scores, an anchor-based criterion that is appropriate to populations such as this one: adults who participate in education for self-management of chronic illness and who have a wide variety of diagnoses and multimorbidities. Such a study would require an index of anxiety that has intuitive meaning, is relatively strongly correlated with HADS anxiety scores, is measured independently of the HADS, and is easier to interpret than the HADS. It would also require a similar index of depression.

Further analysis of the decay of impact might benefit from techniques developed specifically for analyzing longitudinal data [51], or from structural equation modeling and multilevel modeling of individual growth curves [19], [52].

### Limitations, and Implications for Further Study

One limitation of this study is that we had no information about why some participants did not return one or more of the follow-up questionnaires (i.e., drop outs). With regard to the number of diagnoses and also with regard to depression scores at baseline, those participants did not differ from the participants who returned all of the follow-up questionnaires (Table 4). Their anxiety scores at baseline were higher, but by only 1.05 points, which was 0.24 standard deviations (Table 4). That difference is small by Cohen’s criteria [37]. It is also smaller than both the distribution-based [40], [41] and the anchor-based [45] criteria for a minimal important difference. The drop outs from the present study remain largely unexplained. Nonetheless, it might be possible to increase participation in studies that use postal questionnaires, as the present study did. Techniques for increasing responses to postal questionnaires have been studied, some of them have been found to be effective, and those should be used to maximize participation in long-term follow-up studies [53].

The precision of the estimate of the time of the start of decay is limited by the fact that data were collected only twice during the year that elapsed between the first and last measurements. More frequent measurements would certainly be useful. If the burden on participants is small, then even daily measurements might be possible [54]. The present results suggest that about one third of the participants who have decay of impact and therefore need reinforcement are likely to need it no later than 6 weeks after the end of the workshops.

Another limitation is that we have little information about possible side effects during the program. By the time of the first follow-up measurement, anxiety had substantially worsened in 16% of the participants and depression had substantially worsened in 24% (as noted in the Results section above). Some worsening in mental health might be expected in the natural course of a chronic medical condition. However, in the present study that worsening might also have been due to symptom sensitization or other unwanted events that can be caused by psychotherapeutic and health-education interventions [55], [56]. Of course such events should be recognized and, as much as possible, prevented. This issue is not directly related to the questions of the prevalence, timing, and magnitude of the decay of impact, but it does have important implications. For example, the frequency of side effects could be one index by which programs are evaluated. The possibility of side effects also underscores the importance of studying changes in individuals rather than only in groups. For example, if it were possible to know that a particular patient is at a high risk of experiencing a side effect of an educational program, then the clinician might choose not to refer that patient to the program. In future studies, Linden’s schema for defining and classifying adverse treatment effects [55] could be adapted from its original context of psychotherapy to self-management education for people with chronic medical conditions.

For further understanding of the decay of impact, it will be important to analyze not only data from longitudinal cohort studies such as this one, but also data from randomized trials with a control group. Randomized trials would be useful because they might reveal patterns of change occurring even in the absence of interventions. For example, while a subgroup of those who receive the intervention have improvement followed by worsening (as documented in the present study), those who do not receive the intervention might experience even greater declines, in which case the intervention could be judged to have been beneficial relative to the control. Randomized trials could also clarify the possible role of regression artifacts [57] (the correlations between the scores used to compute the changes shown in Figure 3 were 0.52 for anxiety and 0.59 for depression).

In part because the decay of impact has not been studied before, there is a need for replication, to determine the extent to which these findings can be generalized to other groups of people with chronic illnesses. Four fifths of the participants were women (Table 1), so these results may not apply to groups with high proportions of men. However, the high proportion of women in this study is probably not an important limitation, because most educational programs of this kind enroll many women and relatively few men [58]. Specifically, in 17 studies that, like the present study, focused on self-management of chronic illness [59]–[75], the percentage of women participants ranged from 61.1% [65] to 88.9% [67]. The percentage in the present study was within that range: 80.2%.

One possible explanation of any apparent improvement or worsening is response shift [76]. The concept of response shift has already been applied to outcomes of health-education programs such as this one [77]–[79]. For response shift to account completely for the apparent decay of impact in the present study it would have to have occurred in opposite directions sequentially. When future longitudinal studies are designed, the possibility of response shift should be taken into account, not least because response shift can be desirable in this context [77].

### Conclusions, and Practical Implications Regarding Reinforcements

The magnitude of the decay of impact after education for self-management of chronic conditions can be quantified by using a distribution-based criterion for a minimal important difference in scores on psychometric scales (in this case, the HADS).

To the best of our knowledge, these results show the first measurements of the magnitude and timing of that decay. Following from this first step, future work should focus not only on replication, on comparisons with controls, on response shift, and on possible side effects, but also on identifying predictors of decay.

It is reasonable to assume that the existence of clinically important decay of impact indicates a need for reinforcement (or at least a need for better tests of reinforcements). These findings imply that reinforcement designed to prevent increases in anxiety and depression was needed as early as 3 months after the baseline measurement (which was 6 weeks after the program ended), and that it was needed by about 20% of this program’s participants.

## References

[1] Newman S, Steed E, Mulligan K (2009) Chronic physical illness: self-management and behavioral interventions. Open University Press: The McGraw-Hill Companies, page 72.

[2] Mulligan K, Newman S (2007) Self-management interventions. In: Ayers S, Baum A, McManus C, Newman S, Wallston K, Weinman J, West R, editors. Cambridge handbook of psychology, health and medicine. 2nd ed. Cambridge University Press, page 393.

[3] Expert Patients Programme. Available: http://www.expertpatients.co.uk/. Accessed 14 May 2013.

[4] Chronic Disease Self-Management Program. Available: http://patienteducation.stanford.edu/programs/cdsmp.html. Accessed 14 May 2013.

[5] Foster G, Taylor SJC, Eldridge S, Ramsay J, Griffiths CJ (2007) Self-management education programmes by lay leaders for people with chronic conditions. Cochrane Database of Systematic Reviews 4: Art. No.: CD005108. 10.1002/14651858.CD005108.pub2 17943839

[6] Barlow J, Turner A, Edwards R, Gilchrist M (2009) A randomised controlled trial of lay-led self-management for people with multiple sclerosis. Patient Educ Couns 77(1): 81–89. 19321290 10.1016/j.pec.2009.02.009

[7] Tousman S, Zeitz H, Taylor LD (2010) A pilot study assessing the impact of a learner-centered adult asthma self-management program on psychological outcomes. Clin Nurs Res 19(1): 71–88. 19933878 10.1177/1054773809354290

[8] Franks P, Chapman B, Duberstein P, Jerant A (2009) Five factor model personality factors moderated the effects of an intervention to enhance chronic disease management self-efficacy. Br J Health Psychol 14(Pt 3): 473–487. 10.1348/135910708X360700 PMC2745728 18808733

[9] Jerant A, Moore-Hill M, Franks P (2009) Home-based, peer-led chronic illness self-management training: findings from a 1-year randomized controlled trial. Ann Fam Med 7(4): 319–327. 19597169 10.1370/afm.996 PMC2713168

[10] Gitlin LN, Chernett NL, Harris LF, Palmer D, Hopkins P (2008) Harvest health: translation of the chronic disease self-management program for older African Americans in a senior setting. Gerontologist 48(5): 698–705. 18981286 10.1093/geront/48.5.698 PMC4091666

[11] Siu AM, Chan CC, Poon PK, Chui DY, Chan SC (2007) Evaluation of the chronic disease self-management program in a Chinese population. Patient Educ Couns 65(1): 42–50. 16872789 10.1016/j.pec.2006.04.013 PMC7135159

[12] Riemsma RP, Taal E, Rasker JJ (2003) Group education for patients with rheumatoid arthritis and their partners. Arthritis Rheum 15 49(4): 556–566. 10.1002/art.11207 12910564

[13] Lorig KR, Ritter PL, Laurent DD, Fries JF (2004) Long-term randomized controlled trials of tailored-print and small-group arthritis self-management interventions. Med Care 42(4): 346–354. 15076811 10.1097/01.mlr.0000118709.74348.65

[14] Clark NM (2003) Management of chronic disease by patients. Annu Rev Public Health 24: 289–313. 12415147 10.1146/annurev.publhealth.24.100901.141021

[15] Caplin DL, Creer TL (1977) A self-management program for adult asthma. III. Maintenance and relapse of skills. J Asthma 38(4): 343–356. 10.1081/jas-100000263 11456388

[16] Green LW (1977) Evaluation and measurement: some dilemmas for health education. Am J Public Health 67(2): 155–161. 402085 10.2105/ajph.67.2.155 PMC1653552

[17] Barlow JH, Wright CC, Turner AP, Bancroft GV (2005) A 12-month follow-up study of self-management training for people with chronic disease: Are changes maintained over time? Br J of Health Psychol 10: 589–599. 16238867 10.1348/135910705X26317

[18] Krebs P, Prochaska JO, Rossi JS (2010) A meta-analysis of computer-tailored interventions for health behavior change. Prev Med 51(3–4): 214–221. Epub 2010 Jun 15. 10.1016/j.ypmed.2010.06.004 PMC2939185 20558196

[19] Hennessy M, Bolan GA, Hoxworth T, Iatesta M, Rhodes F, et al (1999) Using growth curves to determine the timing of booster sessions. Structural Equation Modeling 6(4): 322–342.

[20] Barlow J, Turner A, Swaby L, Gilchrist M, Wright C, et al (2009) An 8-yr follow-up of arthritis self-management programme participants. Rheumatology 48(2): 128–133. 19036778 10.1093/rheumatology/ken429

[21] Riemsma RP, Taal E, Rasker JJ (2003) Group education for patients with rheumatoid arthritis and their partners. Arthritis Rheum 15: 556–566. 10.1002/art.11207 12910564

[22] Lorig K, Holman HR (1989) Long-term outcomes of an arthritis self-management study: effects of reinforcement efforts. Soc Sci Med 29: 221–224. 2665110 10.1016/0277-9536(89)90170-6

[23] Lorig K, Ritter PL, Laurent DD, Plant K, Green M, et al (2010) Online diabetes self-management program: a randomized study. Diab Care 33: 1275–1281. 10.2337/dc09-2153 PMC2875437 20299481

[24] Nguyen HQ, Carrieri-Kohlman V, Rankin SH, Slaughter R, Stulbarg MS (2005) Is Internet-based support for dyspnea self-management in patients with chronic obstructive pulmonary disease possible? Results of a pilot study. Heart Lung 34: 51–62. 15647734 10.1016/j.hrtlng.2004.06.005

[25] Glasgow RE, Toobert DJ, Hampson SE, Strycker LA (2002) Implementation, generalization and long-term results of the “choosing well” diabetes self-management intervention. Patient Educ Couns 48: 115–122. 12401414 10.1016/s0738-3991(02)00025-3

[26] Lorig K, Ritter PL, Villa F, Piette JD (2008) Spanish diabetes self-management with and without automated telephone reinforcement: two randomized trials. Diab Care 31: 408–414. 10.2337/dc07-1313 18096810

[27] Park MJ, Green J, Ishikawa H, Kiuchi T (2013) Hidden decay of impact after education for self-management of chronic illnesses: hypotheses. Chronic Illness. 9(1): 73–80. Epub 2012 July 9. 10.1177/1742395312453351 PMC3697900 22777566

[28] Lorig K, Holman H, Sobel D, Laurent D, González V, et al.. (1993) Living a healthy life with chronic conditions. Palo Alto, CA: Bull Publishing Company (textbook).

[29] Japan Chronic Disease Self-Management Association. Available: http://www.j-cdsm.org/. Accessed 14 May 2013.

[30] Lorig K, Holman H, Sobel D, Laurent D, González V, et al. (2000) Living a Healthy Life with Chronic Conditions: Self-Management of Heart Disease, Arthritis, Diabetes, Asthma, Bronchitis, Emphysema & Others. Palo Alto, CA: Bull Publishing Company (textbook). 2^nd^ edition.

[31] Lorig K, Holman H, Sobel D, Laurent D, González V, et al. (2001) Living a Healthy Life with Chronic Conditions: Self-Management of Heart Disease, Arthritis, Diabetes, Asthma, Bronchitis, Emphysema & Others (2^nd^ edition). (Fusae K, Trans.) Tokyo: Nihon Kango Kyokai, (in Japanese).

[32] Implementation Manual for Stanford Self-Management Programs, 2008. Stanford Patient Education Research Center. Available: http://patienteducation.stanford.edu/licensing/Implementation_Manual2008.pdf. Accessed 14 May 2013.

[33] Japan Chronic Disease Self-Management Association’s 13^th^ leader-training course. Available: http://www.j-cdsm.org/info/news/pdf/bosyuyoko-13th_tokyo_2nd.pdf. Accessed 14 May 2013.

[34] Matsudaira T, Igarashi H, Kiuchi H, Kano R, Mitoma H, et al (2009) Factor structure of the Hospital Anxiety and Depression Scale in Japanese psychiatric outpatient and student populations. Health Qual Life Outcomes 17(7): 42. 10.1186/1477-7525-7-42 PMC2687424 19445722

[35] McDowell I (2006) Measuring Health: A Guide to Rating Scales and Questionnaires. Oxford University Press, USA. 3rd edition page 294.

[36] Bjelland I, Dahl AA, Haug TT, Neckelmann D (2002) The validity of the Hospital Anxiety and Depression Scale. An updated literature review. J Psychosom Res. 52(2): 69–77. 10.1016/s0022-3999(01)00296-3 11832252

[37] Cohen J (1988) Statistical power analysis for the behavioral sciences. 2nd edition. Lawrence Erlbaum Associates.

[38] Nolte S, Elsworth GR, Sinclair AJ, Osborne RH (2007) The extent and breadth of benefits from participating in chronic disease self-management courses: a national patient-reported outcomes survey. Patient Educ Couns 65(3): 351–360. Epub 2006 Oct 5. 10.1016/j.pec.2006.08.016 17027221

[39] Wyrwich K, Bullinger M, Aaronson N, Hays R, Patrick D, et al (2005) Estimating clinically significant differences in quality of life outcomes. Qual Life Res 14: 285–295. 15892420 10.1007/s11136-004-0705-2

[40] Norman GR, Sloan JA, Wyrwich KW (2003) Interpretation of changes in health-related quality of life: the remarkable universality of half a standard deviation. Med Care 41(5): 582–592. 12719681 10.1097/01.MLR.0000062554.74615.4C

[41] Norman GR, Sloan JA, Wyrwich KW (2004) The truly remarkable universality of half a standard deviation: confirmation through another look. Expert Rev Pharmacoecon Outcomes Res. 4(5): 581–585. 10.1586/14737167.4.5.581 19807551

[42] Jaeschke R, Singer J, Guyatt GH (1989) Measurement of health status. Ascertaining the minimal clinically important difference. Control Clin Trials 10(4): 407–415. 2691207 10.1016/0197-2456(89)90005-6

[43] Wyrwich KW, Norquist JM, Lenderking WR, Acaster S (2013) Methods for interpreting change over time in patient-reported outcome measures. Qual Life Res 22(3): 475–483. Epub 2012 Apr 17. 10.1007/s11136-012-0175-x 22528240

[44] Cappellery JC, Bushmakin AG (2013) Interpretation of patient-reported outcomes. Stat Methods Med Res Epub ahead of print 2013 Feb 19. 10.1177/0962280213476377 23427226

[45] Puhan MA, Frey M, Büchi S, Schünemann HJ (2008) The minimal important difference of the hospital anxiety and depression scale in patients with chronic obstructive pulmonary disease. Health Qual Life Outcomes 2(6): 46. 10.1186/1477-7525-6-46 PMC2459149 18597689

[46] Guyatt GH, Berman LB, Townsend M, Pugsley SO, Chambers LW (1987) A measure of quality of life for clinical trials in chronic lung disease. Thorax 42(10): 773–778. 3321537 10.1136/thx.42.10.773 PMC460950

[47] Bennette C, Vickers A (2012) Against quantiles: categorization of continuous variables in epidemiologic research, and its discontents. BMC Med Res Methodol 29(12): 21. 10.1186/1471-2288-12-21 PMC3353173 22375553

[48] Barth J, Martin CR (2005) Factor structure of the Hospital Anxiety and Depression Scale (HADS) in German coronary heart disease patients. Health Qual Life Outcomes 16(3): 15. 10.1186/1477-7525-3-15 PMC555847 15771778

[49] Guyatt GH, Osoba D, Wu AW, Wyrwich KW, Norman GR (2002) Clinical Significance Consensus Meeting Group. Methods to explain the clinical significance of health status measures. Mayo Clin Proc 77(4): 371–383. 11936935 10.4065/77.4.371

[50] McLeod LD, Coon CD, Martin SA, Fehnel SE, Hays RD (2011) Interpreting patient-reported outcome results. Expert Rev Pharmacoecon Outcomes Res 11(2): 163–169. 21476818 10.1586/erp.11.12 PMC3125671

[51] Kenny D, Zautra A (1995) The trait-state-error model for multiwave data. J Consult Clin Psychol 63: 152–159. 10.1037//0022-006x.63.1.52 7896990

[52] McArdle JJ, Ferrer-Caja E, Hamagami F, Woodcock RW (2002) Comparative longitudinal structural analyses of the growth and decline of multiple intellectual abilities over the life span. Dev Psychol 38(1): 115–142. 11806695

[53] Edwards PJ, Roberts I, Clarke MJ, DiGuiseppi C, Wentz R, et al (2009) Methods to increase response to postal and electronic questionnaires (Review). Cochrane Database of Syst Rev 3: 1–12. 10.1002/14651858.MR000008.pub4 PMC8941848 19588449

[54] Gwaltney CJ, Shiffman S, Balabanis MH, Paty JA (2005) Dynamic self-efficacy and outcome expectancies: prediction of smoking lapse and relapse. J Abnorm Psychol 114(4): 661–675. 16351387 10.1037/0021-843X.114.4.661

[55] Linden M (2012) How to Define, Find and Classify Side Effects in Psychotherapy: From Unwanted Events to Adverse Treatment Reactions. Clin Psychol Psychother Doi:10.1002/cpp.1765. Epub ahead of print 2012 Jan 18. 10.1002/cpp.1765 22253218

[56] Muschalla B, Glatz J, Karger G (2011) Cardiac rehabilitation with a structured education programme for patients with chronic heart failure-illness-related knowledge, mental wellbeing and acceptance in participants. [Article in German] Rehabilitation (Stuttg) 50(2): 103–110 Doi:10.1055/s-0030-1265182. Epub 2011 Apr 18. 21503863 10.1055/s-0030-1265182

[57] Campbell DT, Kenny DA (1999) A primer on regression artifacts. New York: The Guilford Press.

[58] Park M, Yamazaki Y, Ishikawa H, Kiuchi T, Green J, et al (2011) Predicting complete loss to follow-up after a health-education program: number of absences and face-to-face contact with a researcher. BMC Med Res Methodol 27 11(1): 145. 10.1186/1471-2288-11-145 PMC3215183 22032732

[59] Fu D, Fu H, McGowan P, Yi-e S, Lizhen Z, et al (2003) Implementation and quantitative evaluation of chronic disease self-management programme in Shanghai, China: randomized controlled trial. Bulletin of the World Health organization 81: 174–182. 12764513 PMC2572423

[60] Elzen H, Slaets JP, Snijders T, Steverink N (2007) Evaluation of the chronic disease self-management program (CDSMP) among chronically ill older people in the Netherlands. Soc Sci Med 64: 1832–1841. 17355901 10.1016/j.socscimed.2007.02.008

[61] Jordan JE, Haynes K, Livingston JA, Osborne RH (2009) Comparison of the pre- post and transition question assessments in a health education setting. J Clin Epidemiol 62: 642–649. 19108986 10.1016/j.jclinepi.2008.07.019

[62] Barlow JH, Turner AP, Wright CC (1998) Long-term outcomes of an arthritis self- management programme. British Journal of Rheumatology 37: 1315–1319. 9973156 10.1093/rheumatology/37.12.1315

[63] Hass M (2005) Groupp E, Muench J, Kraemer D, Mrummel-Smith K (2005) Chronic Disease Self-Management Program for Low Back Pain in the Elderly. J Manipulative Physiol Ther 28: 228–237. 15883575 10.1016/j.jmpt.2005.03.010

[64] Lorig KR, Ritter P, Laurent DD, Plant K (2006) Internet-Based Chronic Disease Self-Management: A Randomized Trial. Med Care 44: 964–971. 17063127 10.1097/01.mlr.0000233678.80203.c1

[65] Smith L, Bosnic-Anticevich SZ, Mitchell B, Saini B, Krass I, et al (2007) Treating asthma with a self-management model of illness behavior in an Australian community pharmacy setting. Soc Sci Med 64: 1501–1511. 17202024 10.1016/j.socscimed.2006.11.006

[66] Lorig KR, Sobel DS, Stewart AL, Brown BW, Bandura A, et al (1999) Evidence suggesting that a Chronic Disease Self-Management Program can improve health status while reducing hospitalization: a randomized trial. Med Care 37: 5–14. 10413387 10.1097/00005650-199901000-00003

[67] Yip YB, Sit JW, Wong D, Chong S, Chung LH (2008) A 1-year follow-up of an experimental study of a self-management arthritis programme with an added exercise component of clients with osteoarthritis of the knee. Psychol Health Med 13(4): 402–414. 18825579 10.1080/13548500701584030

[68] Damush TM, Weinberger M, Perkins SM, Rao JK, Tierney WM, et al (2003) The long-term effects of a self-management program for inner-city primary care patients with acute low back pain. Arch Intern Med 163: 2632–2638. 14638564 10.1001/archinte.163.21.2632

[69] Lorig KR, Sobel DS, Ritter PL, Hobbs M (2001) Effect of a self-management program on patients with chronic disease. Eff Clin Pract 4: 256–262. 11769298

[70] Swerissen H, Belfrage J, Weeks A, Jordan L, Walker C, et al (2006) A randomized control trial of a self-management program for people with a chronic illness from Vietnamese, Chinese, Italian and Greek backgrounds. Patient Educ Couns 64(1–3): 360–368. 16859871 10.1016/j.pec.2006.04.003

[71] Chan SC, Siu AMH, Poon PKK, Chan CCH (2005) Chronic disease self- management program for Chinese patients: a preliminary multi-baseline study. Int J Rehabil Res 28(4): 351–354. 16319561 10.1097/00004356-200512000-00008

[72] Lorig KR, Ritter PL, Jacquez A (2005) Outcomes of Border Health Spanish/English Chronic Disease Self-Management Programs. Diabetes Educ 31(3): 401–409. 15919640 10.1177/0145721705276574

[73] Gitlin LN, Chernett NL, Harris LF, Palmer D, Hopkins P, et al (2008) Harvest health: translation of the Chronic Disease Self-Management Program for older African Americans in a Senior Setting. Gerontologist 48: 698–705. 18981286 10.1093/geront/48.5.698 PMC4091666

[74] Goeppinger J, Armstrong B, Schwartz T, Ensley D, Brady TJ (2007) Self-management education for persons with arthritis: managing comorbidity and eliminating health disparities. Arthritis Rheum 57(6): 1081–1088. 17665471 10.1002/art.22896

[75] Jerant A, Kravitz R, Moore-Hill M, Franks P (2008) Depressive symptoms moderated the effect of chronic illness self-management training on self-efficacy. Med Care 46: 523–531. 18438201 10.1097/MLR.0b013e31815f53a4

[76] Schwartz CE, Bode R, Repucci N, Becker J, Sprangers MA, et al (2006) The clinical significance of adaptation to changing health: a meta-analysis of response shift. Qual Life Res 15(9): 1533–1550. 17031503 10.1007/s11136-006-0025-9

[77] Osborne RH, Hawkins M, Sprangers MA (2006) Change of perspective: a measurable and desired outcome of chronic disease self-management intervention programs that violates the premise of preintervention/postintervention assessment. Arthritis Rheum 55(3): 458–65. 16739214 10.1002/art.21982

[78] Osborne RH, Elsworth GR, Whitfield K (2007) The Health Education Impact Questionnaire (heiQ): an outcomes and evaluation measure for patient education and self-management interventions for people with chronic conditions. Patient Educ Couns 66(2): 192–201. 17320338 10.1016/j.pec.2006.12.002

[79] Ahmed S, Bourbeau J, Maltais F, Mansour A (2009) The Oort structural equation modeling approach detected a response shift after a COPD self-management program not detected by the Schmitt technique. J Clin Epidemiol. 62(11): 1165–1172. Epub 2009 Aug 6. 10.1016/j.jclinepi.2009.03.015 19664904

